# Exploring Factors Affecting Graduate Students’ Satisfaction toward E-Learning in the Era of the COVID-19 Crisis

**DOI:** 10.3390/ejihpe12080079

**Published:** 2022-08-15

**Authors:** Lubna Ali Mohammed, Musheer A. Aljaberi, Asra Amidi, Rasheed Abdulsalam, Chung-Ying Lin, Rukman Awang Hamat, Atiyeh M. Abdallah

**Affiliations:** 1 Faculty of Social Science, Arts, and Humanities, Lincoln University College, Petaling Jaya 47301, Selangor, Malaysia; 2 Department of Community Health, Faculty of Medicine & Health Sciences, Universiti Putra Malaysia, Serdang 43400, Selangor, Malaysia; 3 Faculty of Medicine & Health Sciences, Taiz University, Taiz 6803, Yemen; 4 Faculty of Nursing & Applied Sciences, Lincoln University College, Petaling Jaya 47301, Selangor, Malaysia; 5 Faculty of Dentistry, Lincoln University College, Petaling Jaya 47301, Selangor, Malaysia; 6 Institute of Allied Health Sciences, College of Medicine, National Cheng Kung University, Tainan 701, Taiwan; 7 Department of Medical Microbiology, Faculty of Medicine & Health Sciences, Universiti Putra Malaysia, Serdang 43400, Selangor, Malaysia; 8 Department of Biomedical Sciences, College of Health Sciences, QU-Health, Qatar University, Doha 2713, Qatar

**Keywords:** e-learning, students’ satisfaction, system quality, course evaluation, student factors, instructor performance, COVID-19

## Abstract

Background: Shifting the educational system from a traditional to an online context during COVID-19 necessitated several considerations to assure students’ satisfaction with e-learning. Aim: This study aims to explore the factors influencing students’ satisfaction with e-learning during the COVID-19 crisis. In particular, it tests multiple mediations, student factors, and system quality between students’ satisfaction and each course evaluation and instructor’s performance. Methodology: In this cross-sectional study, 258 undergraduate and postgraduate students enrolled in online courses at multiple Malaysian universities were recruited using non-probabilistic purposive sampling. Data were collected using a questionnaire that assessed four factors that influenced students’ satisfaction with e-learning during the COVID-19 crisis (i.e., instructor performance, course evaluation, student factors, and system quality) and analyzed using the partial least squares route structural equation modelling (PLS-SEM). Results: The results indicated that the four factors were significantly associated with students’ satisfaction with e-learning during COVID-19. Student factors and system quality were the most factors predicting students’ satisfaction with e-learning. Findings indicate statistically significant relationships between the instructor’s performance, student factors, course evaluation, and system quality on students’ satisfaction. Furthermore, the results depict that both course evaluation and system quality serially mediate the relationship between instructors’ performance and students’ satisfaction. Conclusion: This study finds that improving and enhancing student factors and system quality is critical for students’ satisfaction with e-learning. Furthermore, e-learning platforms should contain new advances of computer-mediated technologies that enable collaboration, which is a critical factor in the success of e-learning systems.

## 1. Introduction

Electronic learning (e-learning) is a form of teaching and learning via computer software using different learning management systems (LMSs) such as Blackboard, Moodle, and WebCT, in which instructors interact with their students, as well as assess them and track their progress through various learning tools [1]. The global pandemic of COVID-19 has posed a significant threat to human life and activities, including education [2,3,4,5,6,7]. Students were unprepared for the transition from traditional to online education and found it difficult to follow the course online which required them to study for longer periods every day, and it negatively affected their academic performance [8,9,10]. In Malaysia, during the Movement Control Order (MCO) period, e-learning became necessary to ensure educational continuity, and more concern was paid to ensure the students’ satisfaction [11,12] as it is the success or failure of any e-learning program [13]. In a recent study, university students showed a high level of preparedness to undergo Online and Distance Learning (ODL) [11].

Although many scholars have assessed the satisfaction level of students within an e-learning environment, due to the overall reliance of the students on e-learning during the crisis of COVID-19, it has become vital to realize the influence of e-learning quality on understanding how satisfied students are with the present content and e-learning quality provided in an online learning environment [14]. However, there is a knowledge gap concerning student satisfaction. Many studies on e-learning have investigated factors that influence students’ satisfaction; the findings are somewhat inconsistent, particularly those related to human factors. In addition, previous literature has focused on various aspects of e-learning satisfaction, but those factors need to be tested against e-learning experiences during crisis times; therefore, this study investigates the different factors affecting students’ satisfaction with the effectiveness of online learning during COVID-19 in Malaysia. Therefore, to fill the above-mentioned gaps, this study pursued to investigate the impact factors influencing students’ satisfaction with e-learning, and mediated the relationship between instructor’s performance (IP) and Students’ Satisfaction (SS).

Students’ satisfaction with e-learning requires designing learning instruction toward building a learning community, which includes various types of interactions [15,16,17,18]. Learners’ satisfaction reflects how they view their learning experience, which is one of the crucial elements to assess the effectiveness of e-learning quality [19]. The quality of service and readiness level of an instructor can affect the course outcomes and student satisfaction [20,21,22]. Recently, Pham et al. [23] showed that e-learning system quality, course and instructor quality, and e-learning administrative and support service quality positively affect university students’ satisfaction and commitment to e-learning. Students’ self-studying behavior and academic achievement were positively influenced by their awareness of the e-learning system [24,25]. The flexibility of e-learning [26] and social presence are other influential factors for student satisfaction. In a study before the COVID-19 pandemic, Al-rahmi et al. [27] reported that Malaysian students were satisfied with using e-learning as it facilitates their studies and motivates them. The findings of Kumar, Saxena, and Baber [14] indicated statistically significant relationships between the e-learning content and e-learning quality and the students’ satisfaction who use e-learning systems during the COVID-19 outbreak. Baber [28] identified the moderating impact of perception about maintaining social distance reduces the effect of social interaction on the effectiveness of online learning during COVID-19.

Assessment is a crucial indicator of the quality of the online program. Instructors review and revise both the course content and the instructional methods based on the online assessment results [29]. Course content, student interaction with course content, and assessment methods in online classes must be developed according to Bloom’s taxonomy [30]. The components of course evaluations must be focused on the effectiveness of teaching and learning. In e-learning, students are independent learners [25], and the instructor’s role is to scaffold, coach the students, and provide them with instant feedback and guidance to accomplish their tasks. Accordingly, the best way to make online learning effective is to focus on three factors: active learning, motivation, and feedback [31]. The instructor’s role in e-learning reflects the constructivism theory’s main principles that believe in the learners’ ability to individually and collectively construct the knowledge they need to solve problems based on their skills. It takes various methods from the learners due to individual differences in their prior knowledge; however, the community as instructors is important in scaffolding them when needed by providing guidance, motivation, and feedback. Student satisfaction is influenced by course assessment and the quality of online courses [32].

After exploring previous studies, three factors related to students’ academic lives were found: (1) social presence in e-learning courses, (2) student–instructor interaction, and (3) students’ awareness of e-learning use. Subsequently, these factors were gathered into one large construct named student factors, as they share the same concept. Social presence influences student satisfaction and online course quality [33,34]. Social presence consists of intimacy and immediacy factors [35]; intimacy refers to the connectedness that communicators display during their interactions, while immediacy refers to the psychological distance between them. In distance education, reciprocal interaction between students and instructors is an essential attribute of a quality learning experience [36], and it influences the level of students’ satisfaction [37,38]. Although institutions are aware of these factors, they are sometimes intermittent or ignored due to unpredictable technical issues, leaving students frustrated and unmotivated to continue their e-learning [39]. Students’ satisfaction was positively associated with the instructor’s degree of success in accomplishing the psychological obligation contract between them and the students; such performance motivates both parties [40,41]. In this study, student–instructor interaction refers to the instructors’ efforts in building a mutual interpersonal relationship with students. This study set out to answer the following questions: what are the factors that influence student satisfaction in an e-learning environment? What is the relation between student factors and student satisfaction in the e-learning environment?

## 2. Literature Review

COVID-19 has had an impact on students’ lives in different aspects: well-being; behavior, and learning. In a cross-cultural study conducted by Cifuentes-Faura et al. [42], it was found that student well-being has deteriorated in all the countries studied (Oman, Spain, Nigeria, and Cambodia). When it comes to their studies, students put more effort into their academics than in pre-pandemic times. In addition, students experience job insecurity and receive less social assistance. Because of COVID-19’s safety precautions, online learning has become a useful and practical instrument for curriculum delivery around the world; however, for online teaching and learning, limited access to the internet the is considered the most obvious challenge in some countries [43,44]. Nevertheless, according to Elshami et al. [45], students benefit from online learning for a variety of reasons, including simple access to knowledge, proper content distribution, content standardization, individualized training, self-pacing, interaction, and enhanced convenience, according to the research. Even though online learning is the only option during the COVID-19 pandemic, students’ satisfaction is critical to a successful and effective learning process. Satisfaction among students is defined as an attitude arising from an assessment of the educational experience, facilities, and services [46]. Accordingly, Student satisfaction measurement in online learning is a crucial aspect of successfully developing educational processes for institutions, instructors, and students. Various researchers looked into students’ perceptions of e-learning during the lockdown period, as well as its impact on their learning satisfaction. However, there is a lack of comprehensive characteristics on e-learning satisfaction during pandemics. In this study, three categories of factors were selected: (1) social presence in e-learning courses, (2) student–instructor interaction, and (3) students’ awareness of e-learning use. Subsequently, we gathered these factors into one large construct named student factors, as they share the same concept. Social presence influences student satisfaction and online course quality [47]. The ability to communicate with others virtually is measured by social presence.

Nasir [47] asserted that students who declared a relatively high level of satisfaction were more likely to report a high level of interaction with their peers in online conversation and a high level of social presence. Essentially, social presence seemed to contribute the most to predicting the level of course satisfaction amongst the students. To achieve social presence, the structure should allow for open communication, group cohesion, and useful personal connections. It also refers to a community of inquiry’s ability to allow students to express themselves socially and emotionally using any means of communication available [48]. Student–instructor interaction refers to the instructors’ efforts in building a mutual interpersonal relationship with students.

According to Muzammil et al. [49], the interaction among students, the interaction between students and teacher, and the interaction between students and content have a positive effect on student engagement. The findings also demonstrated that student engagement has a positive influence on student satisfaction. The study of Flanigan et al. [50] showed that intuitions into how instructors approach the rapport-building process with students in online learning settings can be utilized as a framework for assisting instructors to make rapport-related assessments in their online classes. It was asserted that interaction between members influences their insight and experiences of online groups. Particularly, as by-products of social networks, the formation of a sense of community in e-learning platforms are strongly associated with the interactions between members. The finding of this study demonstrated that the perceived ease of use and social influence significantly affected students’ behavioral intention (BI) in online learning [51].

Online learning systems have been developed to empower a student to connect and communicate with instructors and other students. An essential means of keeping students’ sense of community is to keenly take part in online communications [52]. Interpersonal interaction in e-learning can be classified into two categories: student–instructor and student–student. Students may develop a sense of belonging and importance about themselves if they can communicate freely with the instructor and receive active and polite feedback from the instructor via the e-learning system. Students may perceive a sense of closeness with other students and have an impact on what happens within the e-learning environment if they can easily and rapidly exchange knowledge with other students and effectively collaborate with them [53].

Students’ satisfaction was positively associated with the instructor’s degree of success in accomplishing the psychological obligation contract between them and the students; such performance motivates both parties [40,41] and clears any miscommunication that might take place in the e-learning environment [54]. In addition, other factors such as student achievement, the quality of e-learning opportunities provided to students, a lack of authentic, immediate activities, the availability of learning resources [55], and some psychological factors were found to influence the students’ satisfaction even though the students are satisfied with the instructor and the course content [56]. In this study, student–instructor interaction refers to the instructors’ efforts in building a mutual interpersonal relationship with students.

Zakariah et al. [57] studied students’ awareness of e-learning in higher learning institutions in Malaysia and found that the students are interested in this new technology and willing to embrace e-learning. At the same time, technology automaticity and efficacy were found to be a predictor of student satisfaction [58,59]. The findings of Zakariah et al. [57] showed that e-learning can be more easily accepted if it can deliver at least the same learning experience based on existing educational styles and an interactive learning environment. Students’ acceptance is also high, which shows that respondents embrace e-learning as one technique of teaching and learning. E-Learning in higher learning Institutions aims to provide students greater autonomy regarding the point in time, the content, and the method by which they learn by providing on-demand learning, which eliminates the barriers of time and distance. Okpechi et al. [60] investigated awareness and use of e-learning resources for the acquisition of counselling content among undergraduate would-be counsellors; the study’s findings revealed interclass, inter-school, and inter-gender differences concerning both awareness of accessibility and the use of e-learning sources.

### Model Development

E-learning system quality can be studied as the quality of the e-learning website and is related to the capacity of hardware and software used to meet online teaching and learning demands. Universities that provide e-learning services must ensure that the software and hardware used in the e-learning system are up to date and interoperable for the system to run smoothly and reliably. The e-learning system quality was the most important component of e-learning service quality [23]. Based on the perspectives of Korean and American students, the quality of online support services was found to be highly connected with the acceptance of online learning and student satisfaction [61]. Goh, Leong, Kasmin, Hii, and Tan [15] examined three students’ e-learning experiences that determine learning success and satisfaction in Malaysia: course design, instructor interaction, and peer interaction. According to Machado-Da-Silva et al. [62] system quality, information quality, and quality of service are the most important quality attributes of e-learning services. Focusing on these aspects, it was hypothesized that the system quality (SQ) positively influences students’ satisfaction (SS) with e-learning.

After exploring previous studies, three factors were found to be related to student academic life. Subsequently, these factors were gathered into one large construct named student factors, as they share the same meaning and concept [63]. Teaching and social presences are two major support mechanisms in online learning settings that can account for socio-contextual variables regarding students’ basic psychological needs and satisfaction. Social presence indicates one’s capability to interact with others virtually. It serves as a predictor and is associated with program satisfaction; students with a high degree of social presence are expected to have a high level of course satisfaction [64]. In online agriculture education courses, students’ opinions of the learning environment, social presence, and satisfaction were evaluated. Social presence and the learning environment were shown to account for 26% of the variation in student satisfaction [65]. Accordingly, it was hypothesized in this study that social presence is positively associated with student satisfaction in e-learning.

Interaction is considered one of the very crucial elements in distance education due to the isolation of instructors and students. Student–instructor interaction is described as two-way communication between an instructor and students [66]. In the present study, student–instructor interaction refers to the instructors’ efforts in building a mutual interpersonal relationship between them and their students. Different constructs were examined based on the student perception level. Kuo et al. [67] asserted that student–instructor interaction is identified as the strongest predictor that is substantially associated with student satisfaction. According to Giray [44], the lack of direct communication and involvement between instructors and students is the greatest predictor among online students. Even though increased access to open educational resources and digital media provides students more opportunities to access and expand their knowledge, students require instructor assistance to comprehend different representations of concepts and conceptual understanding of a discipline. Accordingly, this work advances the hypothesis of the positive association of student–instructor interaction with student satisfaction.

Students’ awareness of e-learning usage effectively affected students’ satisfaction with e-learning systems. Students’ awareness refers to students’ attitudes about e-learning. It is affected by several factors such as gender, learning style, and self-efficacy [68]. Students show certain positive or negative attitudes towards any new technology, and these attitudes directly influence students’ behavior regarding the use of the technology. Zabadi and Al-Alawi [69] discovered that the impact of gender, technology usage, and skills are statistically significant on awareness of using e-learning by students. Olum et al. [70], who evaluated the awareness, attitudes, preferences, and challenges to e-learning among undergraduate medicine and nursing students at Makerere University, Uganda, found that 96% of students have heard of e-learning, 17% (*n* = 37) had never browsed or used academic websites or applications, over 60% of the participants needed further training to use e-learning effectively, and up to 75% preferred a hybrid teaching technique. According to the majority of students, e-learning may be used to share learning materials, lectures, revisions, and conversations. Vate-U-Lan [71] identified the relationship between online students’ awareness regarding e-learning on social media sites and their overall satisfaction. It was discovered that students with prior e-learning experience had good attitudes toward online learning and were satisfied with their lives. Accordingly, the authors of this study expected the students’ awareness of the use of E-Learning to be positively associated with student satisfaction.

The instructor’s performance refers to the instructor’s availability to scaffold the students on their tasks and stimulate and treat them equally in the current study. The instructor’s role in e-learning reflects the main principles of the constructivism theory, which assumes students’ ability to construct knowledge both individually and collectively under the instructor’s scaffold, guidance, motivation, and feedback. Bair and Bair [72] indicated that the role of the instructor is an important factor affecting the satisfaction of students. Personal contact between students and instructors is one of the most important aspects of determining a student’s perception of their learning satisfaction. In a survey of online students, however, the most significant component impacting student satisfaction was found to be teacher to student interaction rather than student to student connection [73]. The feedback from the instructor on class activities submitted work and communication that keeps students informed on matters relevant to their learning. In another study using data from 397 responses and structural equation modelling, they discovered that instructor feedback, student self-motivation, degree of communication, and instructor knowledge and assistance were some of the reasons substantially associated with student satisfaction. Most important was that instructor feedback was considerably related to the success of learning outcomes, even in weakly constructed web content design [74]. Accordingly, the instructor’s performance (IP) was hypothesized to positively influences student factors (SFs).

In this study, course evaluation refers to the use of an e-learning system to administer online assignments, continuous assessment tests, and final exams. Because course evaluation is such an important part of assessing learning objectives, it must be practicable, relevant, accurate, and consistent with both the objectives and the course content. Offering evaluation and feedback instantly to students can affect students’ usage and acceptance of the e-learning system. Some lecturers failed to set up online exams and self-assessment exams in their classes, which led to weak performance in the evaluation of the students [75]. The study by Almaiah and Alyoussef [76] revealed that course evaluation has a significant positive impact on system quality and the actual use of e-learning systems suggesting that when the course evaluation is fundamental the e-learning system offers online examination evaluation with instant feedback, this encourages the students to use the e-learning system and effect system quality. It was also found that course evaluation is influenced by the instructor performance which denotes that the instructor’s flexibility and smoothness in delivering content, supporting, and assessing the students, influence their perception of the course quality [77].

Research on the use of student course evaluations have demonstrated a range of uses as indicators of system quality, for improvement of student empowerment, and as instruments to measure educational quality [78]. Accordingly, this study hypothesized that the course evaluation is associated with system quality.

**Figure 1:**
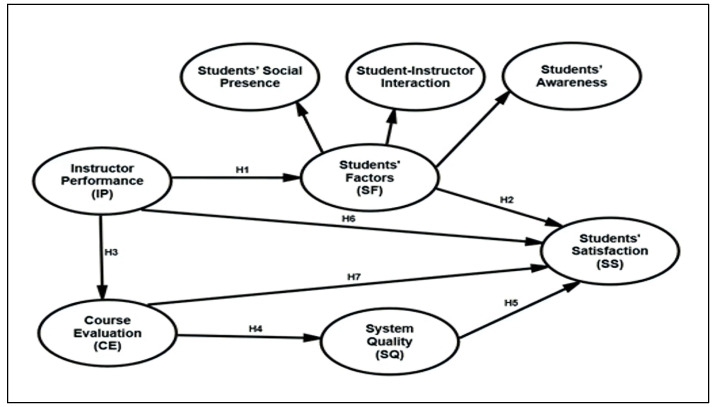
The research model of the study.

Based on the past studies and model development discussed above, the following hypotheses were generated, and the proposed research model of the study is shown in Figure 1, research model of the study:

**Hypothesis** **1** **(H1).** Instructor’s performance (IP) positively influences student factors (SFs).

**Hypothesis** **2** **(H2).** Student factors (SFs) positively influence students’ satisfaction (SS) with e-learning.

**Hypothesis** **3** **(H3).** Instructor’s performance (IP) positively influences course evaluation (CE).

**Hypothesis** **4** **(H4).** Course evaluation (CE) positively influences system quality (SQ).

**Hypothesis** **5** **(H5).** System quality (SQ) positively influences students’ satisfaction (SS) with e-learning.

**Hypothesis** **6** **(H6).** Instructor’s performance (IP) positively influences students’ satisfaction (SS) with e-learning.

**Hypothesis** **7** **(H7).** Course evaluation (CE) positively influences students’ satisfaction (SS) with e-learning.

**Hypothesis** **8** **(H8).** Instructor’s performance (IP) positively and indirectly influences system quality (SQ) via course evaluation (CE).

**Hypothesis** **9** **(H9).** Instructor’s performance (IP) positively and indirectly influences students’ satisfaction (SS) with e-learning via course evaluation (CE).

**Hypothesis** **10** **(H10).** Instructor’s performance (IP) positively and indirectly influences students’ satisfaction (SS) with e-learning via student factors (SFs).

**Hypothesis** **11** **(H11).** Course evaluation (CE) positively and indirectly influences students’ satisfaction (SS) with e-learning via system quality (SQ).

**Hypothesis** **12** **(H12).** Instructor’s performance (IP) indirectly influences students’ satisfaction (SS) through both the course evaluation (CE) and system quality (SQ). In other words, both course evaluation (CE) and system quality (SQ) serially mediate the relationship between the instructor’s performance (IP) and students’ satisfaction (SS).

The above hypotheses show that there are four objectives in this empirical study:

1. To investigate the factors affecting students’ satisfaction with e-learning during the COVID-19 crisis;
2. To test multiple mediations: (a) student factors and (b) system quality between course evaluation, instructor’s performance, and student satisfaction;
3. To test the mediation of course evaluation between instructor’s performance and system quality with student satisfaction;
4. To examine serial mediation between the instructor’s performance and student satisfaction via course evaluation and system quality.

## 3. Methodology

### 3.1. Research Design and Setting

A cross-sectional study was conducted in Malaysia, specifically among the students joining online courses in Malaysian universities. Creswell [79] defined quantitative research as a means for testing objective theories by examining the relationship among variables. Therefore, the current study aimed to analyze the complex interrelationships among a series of variables with mediation. Student satisfaction was an exogenous variable, while student factors and system quality were endogenous variables, which are multiple mediators between instructor performance, course evaluation, and students’ satisfaction, (Figure 1). In addition, student factors were the endogenous variable for instructor performance and course evaluation, which serves as a single mediation between instructor performance and system quality.

### 3.2. Sample Size

A sample size of 258 was deemed adequate for applying partial least squares structural equation modelling PLS-SEM (e.g., smartPLS) to address the research objectives. Hair et al. [80] and Swan [81] indicated that PLS-SEM can be used even in research with less than 100 samples. Using the calculation for a priori sample size for structural equation models (https://www.danielsoper.com/statcalc/calculator.aspx?id=89) (accessed on 1 April 2020), the effect size required is at least 0.50 as a large size [82]. The desired statistical power level is 0.90 as a strong level. The number of latent variables was eight hypothesized constructs, while the number of observed variables was 46 items. The probability level appropriate for SEM is 0.001 and below. By compensating these parameter values in the formula of a priori sample size for structural equation models, the appropriate sample size for the current design was 166 cases as the minimum recommended. The sample size of 258 in this research is considered optimal for PLS-SEM. Moreover, statisticians [83] have considered a sample size of at least 200 cases adequate for conducting structural equation modelling.

### 3.3. Sampling and Procedures

Two hundred and fifty-eight undergraduate and postgraduate students studying courses using different LMSs such as Blackboard, Moodle, and WebCT in different universities in Malaysia agreed to participate in the current study. Non-probabilistic purposive sampling was used to recruit the participants. The link to the online instrument was sent to the participants’ emails and Facebook during the COVID-19 outbreak. Table 1 shows the frequency and percentage for each factor related to demographic variables. The number of males (79, 30.6%) was less than that of females (179, 69.4%), while single participants (188, 72.9%) were more than married participants (70, 27.1%). Regarding age categories, students between 18 and 24 years old (134, 51.9%) were more than students between 25 and 34 years old (78, 30.2%), while students between 35 and 44 years old had the slightest presence in the sample. Concerning the location of residence, urban students (150, 58.1%) were more than suburban students (85, 32.9%), while rural students (23, 8.9%) had the slightest presence in the sample. Regarding academic status, students with bachelor’s degrees were 155 (60.1%); diploma students, 70 (27.1%); master’s students, 23 (8.9%); followed by doctorate students 10 (3.9%).

### 3.4. Instruments

The questionnaire used in the current study consisted of eight sections: the first section measured the demographic data of the participants, and the other seven measured students’ satisfaction, system quality, three student factors, instructors’ performance quality, and course evaluation. Fifty-four items are shown in (Table S1), and they were adopted from four previous studies [20,24,65,84]. All the constructed items were answered using a five-point Likert scale (5 = strongly agree, 4 = agree, 3 = uncertain, 2 = disagree, and 1 = strongly disagree). The internal consistency of these constructs was satisfactory as reported in the previous studies: student–instructor interaction (five items; alpha = 0.737; [84]), instructor performance quality (four items; alpha = 0.882; [84]), course evaluation (six items; alpha = 0.882; [84], social presence (12 items; alpha = 0.94; [65]), system quality (four items; alpha = 0.680; [20]), students’ awareness toward using e-learning in the educational process (16 items; alpha = 0.70; [24]), and students’ satisfaction (seven items; alpha = 0.89; [65]). In addition to the previous studies which developed and evaluated these instruments [85,86,87,88,89], the current study used advanced analyses to evaluate the psychometric properties of these instruments for each respective factor.

### 3.5. Ethical Approval and Consent to Participate

Ethical approval from the Ethics Research Committee of Lincoln University College was granted, and electronic informed consent was obtained from the participants. The participants were granted the right to withdraw from the study at any time. The principles of anonymity and confidentiality were applied.

### 3.6. Statistical Data Analysis

#### 3.6.1. Descriptive Statistics

IBM SPSS 25 was used to obtain the mean, standard deviation, skew, kurtosis, and reliability for all items of the hypothesized model. Jeffreys’s Amazing Statistics Program (JASP) project was used to calculate omega reliability, which is considered more accurate than other types of reliabilities [90].

#### 3.6.2. PLS–SEM

Variance-based structural equation modeling (i.e., PLS-SEM) with SmartPLS 3.0 [91] is used to validate the instrument or items in a stage of the measurement model, providing evidence of convergent (e.g., high loading, Average Variance Extracted (AVE) types of reliability), and discriminate validity (Fornell–Larcker Criterion and Heterotrait–Monotrait ratio of correlations) [92,93,94,95,96]. Afterwards, another structural model was tested to verify the direct and indirect hypotheses explained earlier. The Monte Carlo method using the R project was additionally performed to test mediated variables [97]. After obtaining the path coefficients for A and B paths, the standard deviations for paths A and B were squared following procedures explained by Selig and Preacher (http://quantpsy.org/medmc/medmc.htm) (accessed on 6 June 2020).

## 4. Results

### 4.1. Descriptive Statistics Results

Table S1 illustrates the descriptive statistics of the instruments used in the current study for predictive factors affecting satisfaction with e-learning among students in Malaysian universities during the COVID-19 crisis. The means of all factors are centered on four scores with a standard deviation of less than 1, which means that all participants agreed on the given items. Skewness (≤−/+2) and kurtosis (≤−/+2) indicated that all items of the hypothesized model were normally distributed [98]. Both Cronbach’s Alpha (α) and McDonald’s omega (ω) for each item in each factor outperformed the given criteria (≥0.70) [83,90,92], which confirmed that each item consistently and positively assesses its corresponding factors. In brief, all items of the hypothesized model were suitable for subsequent analysis without any doubts concerning the obtained results.

### 4.2. Measurement Model Analysis Results

The first procedure was to verify how items loaded onto their corresponding factors. This is to examine if the structure of the hypothesized model of student satisfaction fits reality via the collected data. The second procedure was to determine the constructs’ internal consistency and reliability. The final procedure in determining the model structure was to calculate convergent and discriminant validity.

#### 4.2.1. Convergent Validity

**Figure 2:**
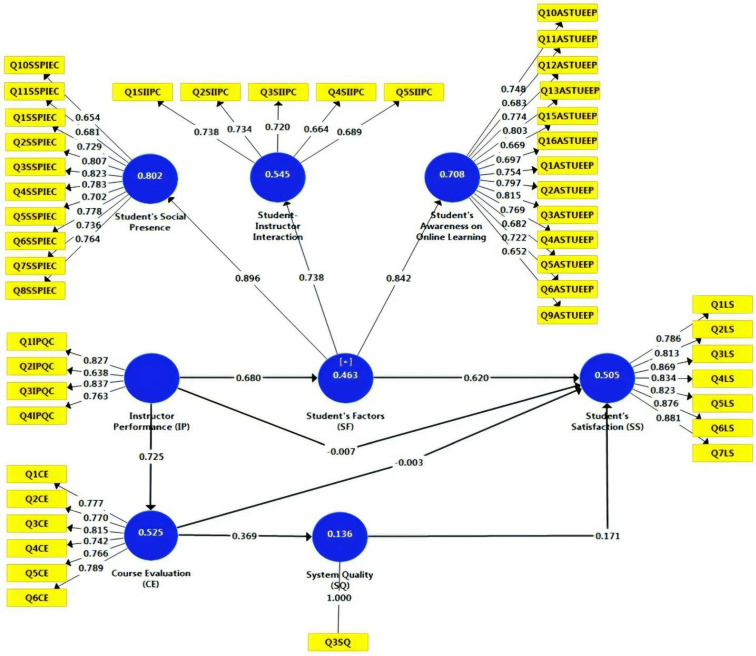
Hypothesized model of study.

The external loadings for factors predicting student satisfaction are shown in Table S2 and Figure 2. The high external loadings supported the convergent validity of measurement validity of factor predicting statistical satisfaction. Results presented in Table 2 confirm the external loadings’ statistical significance in that T-statistics is above its critical ratio of 1.964 and *p*-value ≤ 0.05. All loadings in the hypothesized model were above 0.70, indicating ideal loading on the associated constructs except for a few items loaded at a good rate (above 0.60). These items are Q10SSPIEC, Q11SSPIEC in students’ social presence, Q4SIIPC in the student–instructor interaction, Q11ASTUEEP, Q15ASTUEEP, Q5ASTUEEP, and Q9ASTUEEP in students’ awareness about online learning. In brief, all items had significant contributions in explaining the underlying factors they express.

Reliability outcomes were above the acceptance criteria of 0.70 in the four methods used (Cronbach’s Alpha, rho-A, composite reliability, and McDonald’s ω). Convergent validity is evaluated as a measure of commonality, which refers to the degree of construct that should be related. Convergent validity is calculated by the average variance extracted (AVE) scores, and measures above 0.50 are acceptable [83,92,95,99]. Table 2 shows that the convergent validity measures for predictive factors affecting students’ satisfaction with e-learning during the COVID-19 crisis fall above the 0.50 cut-off for acceptable evaluation. Thus, AVE is advanced evidence for validity for the hypothesized model obtained from average squared factor loadings.

#### 4.2.2. Discriminant Validity

Discriminant validity estimates how the constructs differ in the correlation and whether the factors load mainly on a single construct. Discriminant validity is measured using the Fornell–Larcker Criterion and Heterotrait–Monotrait ratio of correlations (HTMT) [93,94,96].

##### Fornell–Larcker Criterion

Fornell–Larcker Criterion identified that the square root of the AVEs for all constructs of the students’ satisfaction model on the diagonals as represented by the bolded values were higher than the correlations between constructs (corresponding row and column values) (Table 3). This means that the factors are strongly related to their corresponding indicators compared with other model constructs [94]. This suggests good discriminant validity [92,95]. Additionally, the correlations among all constructs were less than 0.85; thus, the discriminant validity of the model’s constructs was achieved [83,99].

##### Heterotrait–Monotrait Ratio of Correlations (HTMT)

The Heterotrait–Monotrait ratio (HTMT) also measures discriminant validity, examining the correlations within construct indicators and indicators across constructs. The upper threshold for this study was 0.9, as suggested for models where the constructs are similar in concept [96]. The HTMT value across the instructor’s performance (IP) and course evaluation (CE) was 0.877. The same procedures calculate the rest of the HTMT for other relationships in the hypothesized model. However, the SmartPLS-calculated HTMT, as presented in Table 4, is automatically based on the given formula [96].

All the HTMT values for the predictor factors were lower than the threshold value of 0.90, demonstrating that discriminant validity was determined. The exception was made for the hierarchical construct order that contained three factors: student–instructor interaction, students’ awareness of online learning, and students’ social presence with the hierarchical construct order labelling the student factors. These three factors are components of student factors (SFs). Subsequently, it is expected that the results are more than the threshold value of 0.90 concerning hierarchical construct orders as each of the three factors is a student factor (SF).

### 4.3. Structural Model

#### 4.3.1. Direct Hypotheses

##### Instructor’s Performance (IP)→Student Factors (SFs)

H1: Instructor’s performance (IP) positively influences student factors (SFs). The results indicated that H1 was statistically significant (*T*-value = 15.342, above critical value = 1.964, and *p* = 0.000, *p* ≤ 0.05) (Table 5). Consequently, H1 is supported, demonstrating a positive relationship between IP and SF. The higher level of IP, the more the SF. The direct path coefficient (β) was 0.680, ranging between the LL (0.583) and UL (0.756) as the stability of the result. The effect of IP on SF was around 46%.

##### Student Factors (SFs)→Student Satisfaction (SS)

H2: Student factors positively influence students’ satisfaction with e-learning. The results indicate that H2 is statistically significant (*T*-value = 6.108, above critical value = 1.964, and *p* = 0.000, *p* ≤ 0.05) (Table 5). Consequently, H2 is supported, demonstrating the positive relationship between SF and SS. This means the higher the level of SF, the more the SS. The direct path coefficient (β) was 0.620, ranging between the LL (0.395) and UL (0.792) as the stability of the result. The effect of SF on SS was around 38%.

H3, H4, and H5 were statistically significant (*T*-value = 19.365/4.988/2.999, above critical value = 1.964, and *p* = 0.000, 0.000 and 0.003, *p* ≤ 0.05) (Table 5 and Figure 2). Their direct path coefficients (β) were 0.725, 0.369, and 0.171. In contrast to this, H6 and H7 were statistically insignificant (*T*-value = 0.082/0.031, below critical value = 1.964, and *p* = 0.935 and 0.975, *p* ≥ 0.05) (Table 5). Their direct path coefficients (β) were negligible.

#### 4.3.2. Single Mediation

H8: Instructor’s performance (IP) positively and indirectly influences system quality (SQ) via course evaluation (CE).

**Figure 3:**
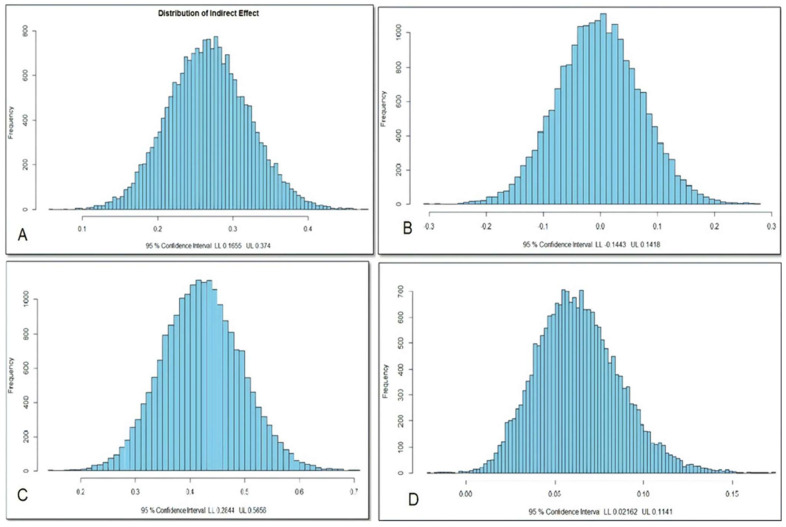
(**A**) Monte Carlo method for distribution of indirect effect for H8, (**B**) Monte Carlo method for distribution of indirect effect for H9, (**C**) Monte Carlo method for distribution of indirect effect for H10, and (**D**) Monte Carlo method for distribution of indirect effect for H11.

The results indicate that H8 is statistically significant (*T*-value = 4.790, above critical value = 1.964, and *p* = 0.007: *p* ≤ 0.05) (Table 6). Consequently, H8 is supported, demonstrating the positive relationship between IP and SQ via CE. This means that CE transmits the effect of IP to SQ effectively. The direct path coefficient (β) was 0.268, ranging between the LL (0.164) and UL (0.372) as the stability of the result. The effect of IP on SQ via CE was around 7%. Moreover, the Monte Carlo method for testing mediation illustrates that H8 is statistically significant as its lower limit (LL) (0.165) and upper limit (UL) (0.374), located in the positive pole of the indirect distributional path of H8, and no negative sign, validating the significance of the hypothesis (Figure 3A; Monte Carlo method for distribution of indirect effect for H8).

H9: Instructor’s performance (IP) positively and indirectly influences the students’ satisfaction (SS) with e-learning via course evaluation (CE).

The results indicate that H9 is statistically insignificant (*T*-value = 0.030, below critical value = 1.964, and *p* = 0.976: *p* ≥ 0.05) (Table 6). Consequently, H9 is not supported, demonstrating any positive relationship between IP and SS via CE, which means that the CE does not transmit the effect of IP to SS effectively. Moreover, the Monte Carlo method for testing mediation illustrates that H9 is not statistically significant as the lower limit (LL) (−0.144) and upper limit (UL) (0.141) are located in the negative and positive poles of the indirect distributional path of H9, and there is no negative sign validating the significance of the hypothesis (Figure 3B; Monte Carlo Method for distribution of indirect effect for H9).

H10: Instructor’s performance (IP) Positively and indirectly influences the students’ satisfaction (SS) with e-learning via student factors (SFs).

The results indicate that H10 is statistically significant (*T*-value = 5.242, above critical value = 1.964, and *p* = 0.000: *p* ≤ 0.05) (Table 6). Consequently, H10 is supported, demonstrating the positive relationship between IP and SS via SF. This means SF transits the effect of IP to SS effectively. The direct path coefficient (β) is 0.422, ranging between the LL (0.268) and UL (0.577) as the stability of the result. The effect of IP on SS via SF was around 18%. Moreover, the Monte Carlo Method for testing mediation illustrates that H10 is statistically significant as the lower limit (LL) is (0.283), and the upper limit is (UL) (0.565), which is located in the positive pole of the indirect distributional path of H10 (Figure 3C; Monte Carlo method for distribution of indirect effect for H10).

H11: Course evaluation (CE) positively and indirectly influences students’ satisfaction (Ss) with e-learning via system quality (SQ).

The results indicated that H11 was statistically significant (*T*-value = 2.769, above critical value = 1.964, and *p* = 0.006: *p* ≤ 0.05) (Table 6). Consequently, H11 is supported, demonstrating the positive relationship between CE and SS via SQ. This means SQ transmits the effect of CE to SS effectively. The direct path coefficient (β) is 0.063, ranging between the LL (0.025) and UL (0.113) as the stability of the result. The effect of CE on SS via SQ was around 6%. Moreover, the Monte Carlo method for testing mediation illustrates that H11 is statistically significant as its lower limit is (0.021), and the upper limit is (0.114) (Figure 3D; Monte Carlo method for distribution of indirect effect for H11).

#### 4.3.3. Serial Mediation

The study hypothesized that (H12) IP indirectly influences the student satisfaction (SS) through both course evaluation (CE) and system quality (SQ). Instructor’s performance (IP)→course evaluation (CE)→system quality (SQ)→student satisfaction (SS) (IP)→(CE)→(SQ)→(SS). In other words, both CE and SQ serially mediate the relationship between IP and SS. The results show that this serially indirect hypothesis (H12) is statistically significant (β = 0.034, *T* value = 2.660, above critical value = 1.964, and *p* = 0.008, *p* ≤ 0.05) (Table 6). Furthermore, the lower limit (LL) (0.016) and upper limit (UL) (0.082) of the confidence interval (CI) bias-corrected of boots trapping method are located in the positive direction, confirming the significance of the hypothesis. Consequently, H12 is supported, indicating the positive relationship between IP and SS via CE and SQ, which means that the CE transmits the effect of IP to SQ, which also transfers that effect to SS. The indirect path coefficient/effect (β) is 0.046, which ranges between the LL (0.016) and UL (0.082) as mentioned by the bootstrapping method. This means that 5% of variance from SS is jointly explained by IP via CE and SQ. In other words, the effect of IP on SS via CE and SQ as serial mediation was around 5%.

## 5. Discussion

The evidence presented in Table S2 illustrates that 9 out of the 12 proposed hypotheses, H1, H2, H3, H4, H5, H8, H10, H11, and H12, were supported in the hypothesized conceptual framework, whereas 3; H6, H7, and H9 were not. The discussion of each hypothesis is presented below.

### 5.1. Instructor’s Performance (IP)

The results of this study support H1, which means that an instructor’s performance (IP) positively influences student factors (SFs). This result is similar to the results of previous studies [23,25,31,84]. IP is of paramount importance as e-learning is a new environment for learners [37]. The IP quality in the e-environment facilitates the learning process [31]. The instructor’s role in providing the needed scaffold to students is crucial, although learners are autonomous. However, not all students exhibit a similar level of autonomy [25]; scaffolding and descriptive feedback are necessary for students to acquire knowledge and skills. Therefore, the role of the instructor in e-learning is crucial for the success of the program. In e-learning, the instructor guides, assists, and motivates the students, provides spontaneous feedback and facilitates student interactions for a better learning outcome [31]. The high quality of IP helps motivate and build students’ confidence and autonomy, leading to their satisfaction.

The results also support H3, which indicates the effect of IP on the course evaluation (CE), which is consistent with previous studies’ findings [77]. In the e-learning environment, the instructor transmits knowledge using the relevant instructional design and technology. Hence, the flexibility and smoothness of the instructor in delivering the content, supporting and communicating with students, and assessing them, influences the students’ evaluation of the course quality [32,77]. Consequently, it affects their satisfaction with the entire learning experience. Such findings denote that the quality of the instructor meets students’ expectations.

H6 and H9 were not statistically significant. These results might be due to psychological factors, which vary from one person to another and are not examined in the current study, such as perceived unfairness, inequality, or mistrust. Psychological factors were found to significantly decrease SS [56], even when students were satisfied with both the instructor and course. Moreover, other factors can affect SS and are not examined in this study, such as student achievement at the end of the course, quality of learning opportunities provided to students in online learning, lack of authentic, immediate activities, and learning resources availability [55]. Moreover, these results might be due to Students’ attitudes towards specific subjects. A strong justification might be the challenges of the students to achieve the required level of automaticity and technology efficacy for e-learning, which were found to be SS predictors [58,59]. Instructors must design interactive teaching and facilitate continuous interactions to achieve SS [15,18]. IP in an e-learning environment influences perceived student learning [74], hence it is an essential factor affecting SS and can be a possible justification for this result.

In H8 and H10, IP positively and indirectly influences system quality (SQ) via course evaluation (CE), and students’ satisfaction (SS) with e-learning via student factors (SFs) were supported. These results are consistent with previous studies’ findings [17,20]. The findings suggest that IP is a crucial predictor of satisfaction when it is mediated with proper student factors: students’ presence, student–instructor interaction, and students’ awareness. Moreover, IP is essential and positively affects the system quality of the course when the course evaluation mediates it. The level of readiness of the instructor affects the use of e-learning technology. Joel and Christina, 2018, showed that online system quality was associated with instructor quality [20], and the instructor quality had a positive effect on the course evaluation, as is shown in this study. Therefore, it can be concluded that the relationship between IP and CE positively affects SQ. We observed that student factors and student–student interaction were vital predictors of SS with e-learning courses [17].

The H12 hypothesis, which is the indirect influence of IP on SS through both CE and SQ, was supported in this study. This result is consistent with previous studies [20]. The findings suggest that the association of the three factors IP, CE, and SQ, significantly causes students’ satisfaction with e-learning. Joel and Christina stated that system quality, instructor quality, and service quality influence SS [20]. Because IP significantly affects CE as in H3 [32,77] and affects SQ via CE directly and positively as in H8, CE significantly affects SQ as supported by H4, and SQ positively influences SS as in H5. It is, therefore, logical to find that IP significantly and indirectly affects SS through both CE and SQ.

### 5.2. Student Factors (SFs)

The H2 hypothesis was confirmed by our analysis indicating that student factors (SFs) positively influence students’ satisfaction (SS) with e-learning. This result is similar to those of previous studies [33,34,35,85] which support the significance of social presence in SS. This is because the immediacy of direct communication creates a comfortable and intimate e-learning environment [34]. The importance of social presence compensates for any weaknesses that might occur during the reciprocal interaction mediated by technology. Adopting different integration patterns such as student–student and student–instructor dialogue, gestures, facial expressions, and tone of voice during e-learning makes the communication more satisfactory and comprehensible [33]. Therefore, social communication and reforming unity among the members in the e-learning environment through an emphasis on intimacy and immediacy are crucial to attaining online educational outcomes [35].

The result of H2 is consistent with those of previous empirical research demonstrating the significance of the interaction between students and their instructors and student satisfaction in distance education [15,37,41]. Mutual interaction between students and instructors is vital for e-learning experience quality. The instructor’s immediate feedback and direct support and assistance help in achieving the educational outcomes [38], hence influencing SS. This result is also represented by the students’ need to be related to the psychological contracts with their instructors, which is vital for reciprocal exchange in the e-learning environment [36] and for avoiding miscommunication issues due to the e-learning environment [54]. SS is directly affected by the level of the psychological contract. It helps them build their expectations and judgments on the instructor’s quality, which in turn determines students’ level of satisfaction [41]. Therefore, by providing a strong psychological contract between the students and their instructors, the psychological communication gaps caused by the e-learning environment are bridged [54].

Student satisfaction was also significantly affected by students’ awareness regarding the use of e-learning. This result is consistent with previous studies [24,25]. Student awareness is determined by their attitudes towards the new e-learning environment. The result shows that the students’ awareness of the new environment and satisfaction with their instructor’s performance, as supported in H1, resulted in a good level of self-efficacy, learning tendency, and motivation towards the process. Therefore, as the awareness level regarding the importance of e-learning among students increases, their level of involvement, motivation, and satisfaction with their studies also increases [24,25,26].

### 5.3. System Quality (SQ)

H5 hypothesis, which indicates a positive effect of a system quality (SQ) on SS, was confirmed, consistent with previous studies [20,21,22]. If the system is user-friendly, the learner will use it frequently; therefore, better learning outcome achievements will occur, resulting in SS with e-learning. Technology is the central element of e-learning through which educational processes such as communication, teaching, and assessment are performed. Without excellent SQ, e-learning does not occur.

### 5.4. Course Evaluation (CE)

H4 and H11 were supported, indicating that CE positively influences SQ and positively and indirectly affects SS via SQ. The empirical study results are consistent with previous studies [29,81]. These findings suggest that CE significantly influences SS only when SQ mediates this influence. It was shown that both SQ and CE determine SS [32]. CE is the primary concern of students and determines the level of SS. The more course-related evaluation methods are performed, the higher the level of SS perceived [81]. SQ provides freedom and flexibility for instructors to design various assessments for students [29]; this diversity in evaluation can be smoothly performed only with a high level of SQ.

The H7 hypothesis, which assumed that CE positively influences SS, was not confirmed. This might be because most curricula used for e-learning while collecting this study’s data were designed for face-to-face situations. Course curriculum and content need to be designed by experts according to the subject [81] and mode of delivery to achieve SS. Previous studies have found that SS is influenced by the usability and flexibility of the system, course assessment, and quality of online courses [32]. This means that CE by itself is not a directly significant factor; it must be mediated by other factors such as SQ.

This is the first research investigating students’ social presence, student–instructor interaction, and student awareness as one SF. This study attempts to expand the proposed conceptual framework of students’ satisfaction with e-learning during the COVID-19 crisis by including multiple mediations (SF and SQ) and their effects on SS directly and indirectly, with IP and CE as exogenous factors. The model had the serial mediation between IP and SS via CE as the first mediation and SQ as a sequential mediation.

This study, as any other, has limitations that should be considered in future research: (1) The sample for this study was chosen using a non-random sampling technique. Although the sample size was adequate, it is suggested that future studies use a random sampling technique and a bigger sample size. (2) This study used only a quantitative design; future studies should use a combination of approaches or focus on in-depth qualitative analysis. (3) The results for all participants in all educational levels and different LMS were analyzed, and comparative/case studies for the effective elements of SS with e-learning considering different levels of the students and different LMS are advised to be the focus of future studies. (4) E-learning effective variables on student satisfaction such as service quality, technical support, feedback, and evaluation of services for both instructors and students; and students’ feedback on assessments are advised to be examined in future studies. (5) Finally, the instruments employed in this study were simply examined as part of earlier research investigations, and none of them revealed psychometric characteristics findings for each instrument. However, one of the current study’s strengths is that advanced analyses were utilized to examine the psychometric qualities of these instruments for each respective factor and were included in the results as part of this research. As a result, we recommend that future studies pay closer attention to, and concentrate on, the psychometric features of these measures.

## 6. Conclusions

The current study aims to investigate the factors that influence the students’ satisfaction with e-learning during the COVID-19 crisis. Among the four tested predictive factors (i.e., instructor performance, course evaluation, student factors, and system quality), student factors and system quality were the main factors that significantly influenced student satisfaction; directly and indirectly. The results will be used to revise and update the current instructional design to make it more appropriate for the e-learning environment. The new/revised instructional design must emphasize the presence of the students and instructors and the student–instructor interaction via a smooth, flexible, and user-friendly system. The system must be designed to facilitate the interaction and support the e-learning interactional instructional teaching and learning practices. For example, a cloud-computing e-learning platform may resolve the problems of instability. Specifically, cloud computing resolves the problems of using processor algorithms that improve adaptability, dependability, and scalability. In this regard, the computational load can be minimized and the computational resources can be allocated effectively to resolve the problems of instability in the e-learning system. By achieving a high level of e-learning system quality and the students’ factors, the level of student satisfaction will be significantly improved as found in the results of the current study.

The study’s findings will inform students, instructors, program coordinators, and educational policymakers about the relevance of including the factors discovered to be significant in student satisfaction with e-learning in this study. As a result, e-learning programs must ensure the quality of the instructor and his/her performance in e-learning since the instructor has a direct effect on the students, who are the most important factor in the success of any educational program. The instructor’s presence and ability to create a mutual intrapersonal relationship with students, as well as involving the students in reciprocal communication, affect the degree of perceived e-learning among the students and the level of their presence and participation; all these factors lead to student satisfaction and good academic outcomes. Moreover, the quality of educational courses is determined by evaluation. As a result, instructors, program coordinators, and educational policymakers must construct evaluation tools based on Bloom’s taxonomy, and adopt the active approach to learning and teaching. The course evaluation elements must offer a motivating and interactive e-learning environment that encourages students to learn independently and creatively. Students must receive continual feedback and reciprocal contact from their peers and instructor. The higher the level of course evaluation and teacher performance, the higher the quality of the e-learning system, and hence the higher the degree of SS and learning output.

## Figures and Tables

**Table 1:** Demographics of the present sample.

Variable	Type	Frequency	Percentage
Gender	Male	79	30.6
	Female	179	69.4
Marital Status	Single	188	72.9
	Married	70	27.1
Age categories	18–24 years old	134	51.9
	25–34 years old	78	30.2
	35–44 years old	46	17.8
Living	Rural	23	8.9
	Urban	150	58.1
	Suburban	85	32.9
Academic Status	Diploma	70	27.1
	Master’s	23	8.9
	Doctorate degree	10	3.9
	Bachelor’s degree	155	60.1

**Table 2:** Construct reliability and validity.

	Cronbach’s Alpha ≥0.70	rho_A ≥0.70	Composite Reliability ≥0.70	McDonald’s ω ≥ 0.70	AVE ≥0.50
Course Evaluation	0.868	0.871	0.901	0.869	0.603
Instructor’s Performance	0.769	0.787	0.853	0.776	0.594
Student Factors	0.757	0.770	0.861	0.770	0.676
Student–Instructor Interaction	0.754	0.756	0.835	0.755	0.503
Students’ Awareness of _Online Learning	0.930	0.932	0.939	0.905	0.544
Students’ Satisfaction	0.931	0.937	0.944	0.932	0.708
Students’ Social Presence	0.911	0.915	0.927	0.894	0.559
System Quality					

**Table 3:** Fornell–Larcker Criterion for hypothesized model.

Variables	1	2	3	4	5	6	7	8
Course Evaluation 1	0.777							
Instructor’s Performance 2	0.524	0.771						
Student Factors 3	0.488	0.461	0.822					
Student–Instructor Interaction 4	0.363	0.362	0.544	0.709				
Students’ Awareness of _Online Learning 5_	0.332	0.277	0.708	0.159	0.738			
Students’ Satisfaction 6	0.238	0.227	0.481	0.122	0.625	0.841		
Students’ Social Presence 7	0.332	0.383	0.802	0.277	0.487	0.3721	0.748	
System Quality 8	0.137	0.149	0.225	0.050	0.2666	0.213	0.206	1

**Table 4:** Heterotrait–Monotrait Ratio (HTMT).

	1	2	3	4	5	6	7	8
Course Evaluation (CE)								
Instructor’s Performance (IP)	0.879							
Student factors (SFs)	0.867	0.888						
Student–Instructor Interaction (SII)	0.730	0.770	1.010					
Students’ Awareness of Online Learning	0.640	0.615	0.982	0.465				
Students’ Satisfaction (SS)	0.527	0.538	0.799	0.399	0.839			
Students’ Social Presence (SSP)	0.695	0.733	1.065	0.622	0.755	0.651		
System Quality (SQ)	0.396	0.446	0.536	0.254	0.535	0.468	0.474	

**Table 5:** Parameters of direct hypotheses.

N	Hypotheses	β ≥ 0.15	Standard Deviation	T ≥ 1.946	*p* ≤ 0.05	LL 2.5%	UL 97.5%	Decision	ƞ
H_1_	IP→SF	0.680	0.044	15.342	0.000	0.583	0.756	Supported	0.462
H_2_	SF→SS	0.620	0.101	6.108	0.000	0.395	0.792	Supported	0.384
H_3_	IP→CE	0.725	0.037	19.365	0.000	0.639	0.784	Supported	0.525
H_4_	CE→SQ	0.369	0.074	4.988	0.000	0.225	0.499	Supported	0.136
H_5_	SQ→SS	0.171	0.057	2.999	0.003	0.065	0.277	Supported	0.0292
H_6_	IP→SS	−0.007	0.080	0.082	0.935	−0.178	0.150	Rejected	ne
H_7_	CE→SS	−0.003	0.100	0.031	0.975	−0.210	0.191	Rejected	ne

Ne: negligible; LL: low limits; UL: upper limits; β: path from original sample (O).

**Table 6:** Parameters of single and serial mediation.

Hypotheses	Original Sample	Standard Deviation	T Statistics	*p* Values	LL	UL	Decision
(IP)-> (CE)-> (SQ) H8	0.268	0.056	4.790	0.000	0.164	0.372	Supported
(IP)-> (CE)-> (SS) H9	−0.002	0.073	0.030	0.976	−0.154	0.135	Rejected
(IP)-> (SF)-> (SS) H10	0.422	0.080	5.242	0.000	0.268	0.577	Supported
(CE)-> (SQ)-> (SS) H11	0.063	0.023	2.769	0.006	0.026	0.113	Supported
(IP)-> (CE)-> (SQ)-> (SS) H12	0.046	0.017	2.660	0.008	0.016	0.082	Supported

## Data Availability

The datasets that support the findings of this study are not openly available Data will be made available from the corresponding author upon reasonable academic and research use request.

## Supplementary Materials

The following supporting information can be downloaded at: https://www.mdpi.com/article/10.3390/ejihpe12080079/s1.
