# Supplementary material for: Exploring Factors Affecting Graduate Students’ Satisfaction toward E-Learning in the Era of the COVID-19 Crisis
Source: Eur J Investig Health Psychol Educ. 2022 Aug 15;12(8):1121–42. doi: 10.3390/ejihpe12080079 (PMC9407398; doi:10.3390/ejihpe12080079)
Supplement: Supplementary file 1 [file ejihpe-12-00079-s001.zip › ejihpe-1806779-supplementary.pdf]

Supplementary Materials

Table S1: Descriptive statistics of instrument used in the current study.

Symbols	Statements	M	SD	Skew ≤ 3	Kurt ≤ 3	α ≥.70	Ω ≥.70
**Student–Instructor Interaction*1**							
**Q1SII**	The instructors encouraged me to become actively involved in the course discussions	3.56	.941	-.746	.645	.710	.711
**Q2SII**	The instructors provided me feedback on my work through comments	3.54	1.002	-.831	.405	.686	.690
**Q3SII**	I was able to interact with the instructors during the course discussions	3.76	.861	-.776	.591	.709	.712
**Q4SII**	The instructors treated me individually	3.29	.927	-.365	-.184	.725	.726
**Q5SII**	The instructors informed me about my progress periodically	3.12	.957	-.431	-.351	.716	.720
						.753	.755
**Instructor's Performance Quality*2**							
**Q1IPQ**	Overall, these instructors were effective	3.69	.811	-.741	.979	.689	.704
**Q2IPQ**	The instructors were available for consultation during office hours or by appointment	3.67	.786	-.519	.463	.769	.778
**Q3IPQ**	The instructors stimulated students' learning	3.71	.812	-.728	.773	.663	.673
**Q4IPQ**	The instructors treated all students fairly	3.76	.966	-.983	.850	.717	.726
						.767	.776
**Course Evaluation (CE)*3**							
**Q1CE**	Overall, I have valuable learning experiences from my courses	3.71	.899	-.842	.750	.847	.848
**Q2CE**	The assignments were relevant and useful	3.70	.883	-.838	.855	.845	.846
**Q3CE**	Courses materials were relevant and useful	3.78	.871	-.900	1.034	.837	.838
**Q4CE**	Expectations were clearly stated either verbally or in the syllabus	3.73	.844	-.743	.799	.852	.854
**Q5CE**	The testing and evaluation procedures were fair	3.56	.899	-.888	1.079	.849	.850
**Q6CE**	The workload was appropriate for the hours of credit	3.55	.813	-.436	-.175	.843	.843
						.868	.869
**Students' Social Presence in E-Learning Courses*4**							
**Q1SSPIEC**	The instructor facilitated discussions in the course	3.62	.800	-.871	1.214	.873	.883
**Q2SSPIEC**	I feel comfortable interacting with other participants in the online course	3.45	1.04	-.603	-.126	.868	.879
**Q3SSPIEC**	I feel comfortable participating in the course discussions	3.58	1.02	-.827	.263	.866	.877
**Q4SSPIEC**	I feel comfortable conversing through this text-based medium	3.55	.920	-.689	.533	.868	.879
**Q5SSPIEC**	Computer-mediated communication is an excellent medium for social interaction	3.48	.999	-.725	.235	.875	.885
**Q6SSPIEC**	The instructor created a feeling of an online community	3.45	.933	-.760	.363	.869	.880
**Q7SSPIEC**	I am able to form distinct individual impressions of some course participants even though we communicate online	3.43	.880	-.395	.106	.872	.882
**Q8SSPIEC**	The introductions enabled me to form a sense of online community	3.45	.899	-.624	.263	.869	.880
**Q9SSPIEC**	Discussions using the medium of computer-mediated communication tend to be more impersonal (unfriendly) than face-to-face discussions	3.30	.975	-.206	-.451	.899	.904
**Q10SSPIEC**	I feel my point of view was acknowledged by other participants in the course	3.44	.807	-.378	.524	.875	.886
**Q11SSPIEC**	I feel comfortable introducing myself in the online course	3.42	.968	-.611	.111	.875	.886
**Q12SSPIEC**	Messages in the online course are impersonal	3.24	.948	-.194	-.308	.892	.900

Symbols	Statements	M	SD	Skew ≤ 3	Kurt ≤ 3	α ≥.70	Ω ≥.70
						.885	.894
**System Quality (SQ)*5**							
**Q1SQ**	The system is easy to use	3.65	.947	-.857	.757	.780	.784
**Q2SQ**	The system is user friendly	3.71	.806	-1.08	1.597	.763	.766
**Q3SQ**	The system is easy to learn	3.71	.876	-1.08	1.432	.775	.785
**Q4SQ**	The operation of the system is stable	3.40	.904	-.493	-.169	.816	.819
						.828	.833
**Students' Awareness Towards the Use of E-Learning in Educational Process*6**							
**Q1ASTUEEP**	There is a need now, and in the future for e-learning, and it will become an integral part of the educational process	3.83	.886	-.985	1.605	.869	.895
**Q2ASTUEEP**	E-learning is suitable for what I study	3.33	1.176	-.499	-.611	.865	.893
**Q3ASTUEEP**	E-learning allows you to more effectively organize learning process	3.39	1.065	-.561	-.163	.865	.893
**Q4ASTUEEP**	E-learning allows the use of modern teaching resources	3.79	.908	-.963	1.137	.869	.894
**Q5ASTUEEP**	E-learning increases the level of my ICT competencies	3.73	.948	-.786	.500	.871	.898
**Q6ASTUEEP**	I am ready to be trained (already trained) with using e-learning courses	3.70	.929	-.925	.841	.870	.896
**Q7ASTUEEP**	E-learning is an extra work	3.21	1.055	-.219	-.677	.908	.919
**Q8ASTUEEP**	Use of e-learning elements in full-time educational form is not preferable	3.51	1.003	-.663	-.002	.900	.917
**Q9ASTUEEP**	I prefer using of e-learning elements partially with educational methods	3.56	.933	-.907	.638	.873	.899
**Q10ASTUEEP**	E-learning helps you turn to additional educational material	3.60	.912	-.891	.810	.869	.895
**Q11ASTUEEP**	E-learning enhanced the educational process by enhancing the communication between the teacher and the student	3.40	.941	-.527	.057	.870	.898
**Q12ASTUEEP**	Working with the electronic course is a valuable use of time and useful	3.53	.963	-.576	.014	.868	.894
**Q13ASTUEEP**	The quality of the e-learning course matches today's level of education	3.50	.947	-.607	.298	.866	.893
**Q14ASTUEEP**	E-learning can replace laboratory and practical learning with a lot of practice and lecture	2.90	1.207	-.187	-1.034	.876	.902
**Q15ASTUEEP**	E-learning can be used in all subjects	2.80	1.214	.017	-1.068	.868	.897
**Q16ASTUEEP**	In E-learning the quantity and quality of the knowledge obtained corresponds to the time spent	3.37	.930	-.572	.176	.870	.897
						.881	.905
**Students' Satisfaction (SS)*7**							
**Q1LS**	I am satisfied with this program	3.53	1.048	-.788	.254	.930	.931
**Q2LS**	Distance education is worth my time	3.47	1.102	-.588	-.355	.925	.925
**Q3LS**	I enjoy studying by distance	3.28	1.125	-.443	-.530	.917	.917
**Q4LS**	Distance education is stimulating	3.19	1.016	-.358	-.288	.921	.921
**Q5LS**	Distance education is exciting	3.16	1.057	-.350	-.532	.923	.923
**Q6LS**	I look forward to learning by distance	3.18	1.124	-.398	-.664	.915	.916
**Q7LS**	I prefer distance education	2.98	1.187	-.110	-.897	.915	.915
						.931	.932

M: mean; SD: standard deviation; Skew: skewness; Kurt: kurtosis; α: Cronbach's alpha, ω = McDonald's omega.

Table S2. Parameters and outer loading of hypothesized model

	Original Sample (O)	Standard Deviation (STDEV)	T Statistics (|O/STDEV|)	P Values
**Students' Factors**				
SF-> ASTUEEP_F	0.847	0.019	43.692	<0.001
SF-> SIIPC_	0.730	0.056	12.975	<0.001
SF-> SSPIEC_F	0.881	0.018	50.022	<0.001
**Students' Social Presence**				
Presence -> Q10SSPIEC	0.654	0.043	15.295	<0.001
Presence -> Q11SSPIEC	0.681	0.043	15.870	<0.001
Presence -> Q1SSPIEC	0.729	0.036	20.388	<0.001
Presence -> Q2SSPIEC	0.807	0.026	30.602	<0.001
Presence -> Q3SSPIEC	0.823	0.025	33.073	<0.001
Presence -> Q4SSPIEC	0.783	0.034	22.906	<0.001
Presence -> Q5SSPIEC	0.702	0.043	16.410	<0.001
Presence -> Q6SSPIEC	0.778	0.034	22.791	<0.001
Presence -> Q7SSPIEC	0.736	0.038	19.383	<0.001
Presence -> Q8sspiec	0.764	0.034	22.792	<0.001
**Students' Awareness on Online Learning**				
Awareness -> Q10ASTUEEP	0.748	0.037	20.296	<0.001
Awareness -> Q11ASTUEEP	0.683	0.041	16.585	<0.001
Awareness -> Q12ASTUEEP	0.774	0.035	21.949	<0.001
Awareness -> Q13ASTUEEP	0.803	0.029	27.843	<0.001
Awareness -> Q15ASTUEEP	0.669	0.035	19.214	<0.001
Awareness -> Q16ASTUEEP	0.697	0.051	13.531	<0.001
Awareness -> Q1ASTUEEP	0.754	0.033	22.700	<0.001
Awareness -> Q2ASTUEEP	0.797	0.028	28.364	<0.001
Awareness -> Q3ASTUEEP	0.815	0.030	27.169	<0.001
Awareness -> Q4ASTUEEP	0.769	0.035	21.871	<0.001
Awareness -> Q5ASTUEEP	0.682	0.045	15.277	<0.001
Awareness -> Q6ASTUEEP	0.722	0.038	19.089	<0.001
Awareness -> Q9ASTUEEP	0.652	0.041	15.737	<0.001
**Student–Instructor Interaction**				
Interaction -> Q1SIIPC	0.738	0.035	20.808	<0.001
Interaction -> Q2SIIPC	0.734	0.042	17.509	<0.001
Interaction -> Q3SIIPC	0.720	0.038	18.919	<0.001
Interaction -> Q4SIIPC	0.664	0.051	12.976	<0.001
Interaction -> Q5SIIPC	0.689	0.037	18.464	<0.001
SQ -> Q3SQ	1.000	0.000		
**Instructor's Performance (IP)**				
IP -> Q1IPQC	0.821	0.022	37.405	<0.001
IP -> Q2IPQC	0.643	0.060	10.678	<0.001
IP -> Q3IPQC	0.835	0.023	36.391	<0.001
IP -> Q4IPQC	0.769	0.031	24.517	<0.001
**Course Evaluation (CE)**				
CE -> Q1CE	0.771	0.033	23.203	<0.001
CE -> Q2CE	0.774	0.038	20.623	<0.001
CE -> Q3CE	0.819	0.025	32.189	<0.001
CE -> Q4CE	0.739	0.043	17.216	<0.001
CE -> Q5CE	0.769	0.036	21.387	<0.001
CE -> Q6CE	0.788	0.027	29.326	<0.001
**Students' Satisfaction (SS)**				
SS -> Q1LS	0.786	0.027	29.053	<0.001

SS -> Q2LS	0.813	0.030	27.138	<0.001
SS -> Q3LS	0.869	0.017	49.696	<0.001
SS -> Q4LS	0.834	0.023	36.646	<0.001
SS -> Q5LS	0.823	0.029	27.980	<0.001
SS -> Q6LS	0.876	0.017	50.599	<0.001
SS -> Q7LS	0.881	0.015	60.455	<0.001
